# Reduction of crayfish activity and light avoidance in response to artificial light at night

**DOI:** 10.17912/micropub.biology.001832

**Published:** 2025-09-24

**Authors:** Katelyn Boyle, Abigail Zagar, Catherine Stewart, Johnathan Davis

**Affiliations:** 1 Biology, Wofford College

## Abstract

The prevalence of artificial light at night can alter nocturnal environments and affect animal behavior. We investigated how the presence of artificial light affects the activity of the variable crayfish *Cambarus latimanus* by manipulating light intensities and providing a choice between dark and lighted environments. Crayfish were more active in the absence of artificial light and preferred dark environments even when light intensities were very low (~5 lx). Our results suggest that crayfish may increase sheltering behavior and seek out darkness in the presence of artificial light, potentially increasing shelter competition and reducing contributions of crayfish to ecosystem function.

**Figure 1. Crayfish response to the presence of artificial light at night:**
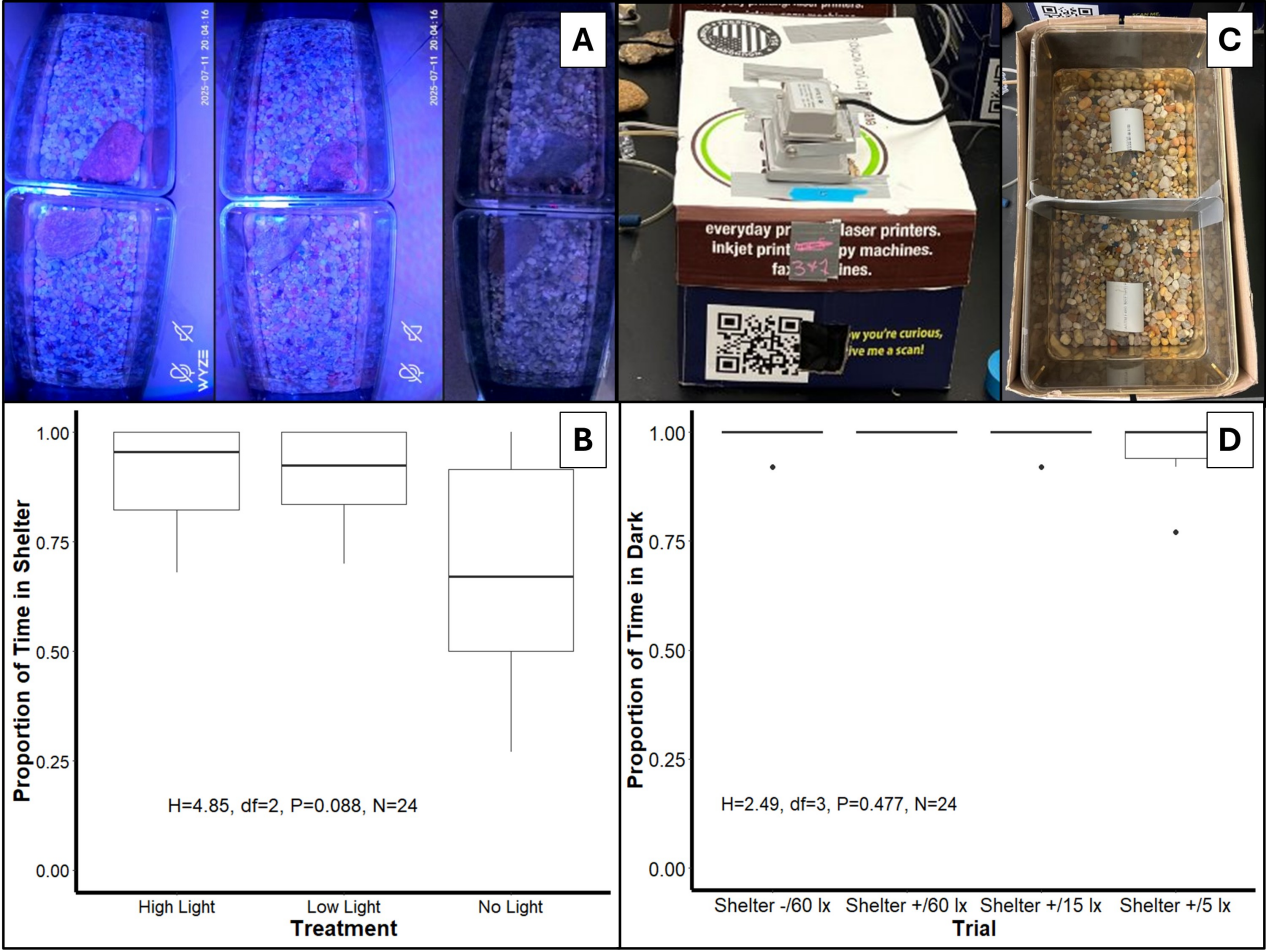
(A) Overhead image captured from cameras mounted above experimental arenas with high (~60 lx, left), low (~15 lx, middle), and no light (right) from Experiment I. (B) Comparison of the proportion of time spent in shelter for high, low, and no light treatments in Experiment I. (C) Image of experimental setup for Experiment II showing darkened arenas enclosed in a box with a light shining on one side of the arena (left) and a divided arena that creates a choice between a side with artificial light and without artificial light (right). (D) Comparison of the proportion of time spent in the dark for 4 trials in Experiment II under the absence (Shelter -) or presence (Shelter +) of shelter and sequentially reduced light intensities (60 lx, 15 lx, and 5 lx).

## Description

Urbanization for commercial, residential, and industrial purposes is characterized by increasing impervious surface cover and decreasing vegetative cover that increases stormwater runoff, flooding, pollution, and sedimentation, leading to a loss of aquatic biodiversity (Dudgeon et al. 2006 Kim et al. 2025; Tang et al. 2025). As urbanization progresses, artificial light at night (ALAN) becomes more prevalent, fundamentally altering nocturnal environments as an added stressor on biological life (Bara and Falchi 2023). However, the effect of ALAN on nocturnal animal behavior has only recently been investigated (Longcore and Rich 2004). An increasing body of research indicates that ALAN affects the timing of various biological activities across many taxa (Gaston et al., 2017) and alters physiology and life history traits (Sanders et al., 2020). It is suggested that artificial light, because of its pervasive impacts to biodiversity and human health, should receive greater focus in the field of global change research (Davies and Smyth 2017).

Crayfish are decapod crustaceans with high imperilment that have the greatest diversity in the southeastern U.S, particularly in the Appalachian mountains (Loughman and Fetzner Jr. 2015). As nocturnal organisms, crayfish are most active at night, conducting activities related to their designation as a keystone species, such as regulating nutrient processing, controlling trophic interactions, and modifying habitats (Reynolds et al., 2013), but the presence of ALAN may limit their nocturnal activity. Exposure of signal crayfish ( *Pacifastacus leniusculus* ) to light pollution reduced nocturnal activity and conspecific interactions (Thomas et al,. 2016). Similarly, New River crayfish ( *Cambarus chasmodactylus* ) and spiny stream crayfish ( *Orconectes cristavarius* ) reduced nocturnal activity when exposed to ecologically-relevant intensities of light under laboratory conditions (Fischer et al., 2020). Exposure can also alter social interactions, particularly agonistic fights (Jackson and Moore 2019). In an investigation of aquaculture conditions, Franke and Horstgen-Schwark (2015) observed decreased nocturnal activity in the presence of light for noble crayfish ( *Astacus astacus* ) *.*

This study investigated the effect of ALAN on crayfish nocturnal activity through a controlled laboratory study using the variable crayfish, *Cambarus latimanus* , a species common to Spartanburg County, South Carolina, one of the fastest growing counties in the U.S. (Tran 2025; U.S. Census Bureau 2024). It was hypothesized that crayfish would be less active when exposed to greater artificial light intensities and prefer darker environments.

To test this hypothesis, two lab-controlled experiments were conducted in which crayfish were exposed to varying light intensities during nocturnal periods. In Experiment I, crayfish in an experimental arena with available shelter were exposed to one of three light intensities (i.e., high light, low light, and no light), and their activity was recorded as either walking, stationary but unsheltered, or sheltering. In Experiment II, crayfish were placed in a divided arena and provided a choice between an environment containing artificial light and a completely darkened environment across four trials. After the first trial that lacked available shelter, shelter was provided, and then light intensity was reduced for each successive trial.

In experiment I, there was no significant difference ( *H(2)* = 4.85, *P* = 0.088) between the proportion of sheltering time among treatments although median sheltering time was reduced and more variable when no artificial light was present. Crayfish were used from two different populations and had two differing lab acclimation times (14 days vs. 2 days), which may have affected sheltering time although there was a non-significant difference in sheltering times between acclimation times ( *H(1)* = 3.42, *P* = 0.064). In addition, there was no significant difference in the proportion of sheltering time among arenas within the same treatment ( *P* = 0.5141).

In experiment II, crayfish spent a significantly greater ( *χ* ^2^ (1,N = 6) = 288.54, *P* < 0.001) amount of time in the dark environment than in the light environment, indicating a preference for dark environments. No significant difference ( *H(3)* = 2.5, *P* = 0.477) was found in the proportion of time spent in darkness between trials, indicating that providing shelter and reducing light intensity did not cause crayfish to choose environments with artificial light more often. No significant tank effects ( *P* = 0.803) were observed across the six arenas used.

Many factors can influence crayfish behavior, including light intensity (Gherardi 2002). In this study, *C. latimanus* activity was reduced in the presence of artificial light in favor of sheltering behavior, and they consistently chose dark environments even under reduced (15 and 5 lx) light intensities. Thus, artificial light may reduce nocturnal activity of this crayfish. Darkened environments at night are hypothesized to provide a less risky environment for crayfish to conduct activities such as movement between habitats, searching and foraging, and agonistic fights. Specifically, nocturnal activity may be an adaptation to minimize predation risk (Holdich 2002), and crayfish, in general, may tend to exhibit greater risk-taking behavior and boldness at night although inter-individual differences can occur in daily activity (Sbragaglia and Breithaupt 2022). Thus, ALAN may drive crayfish to enact more risky behavior in the presence of light, exposing them to greater predation risk, or reduce overall activity in preference for sheltering in burrows. Our results suggest a preference in *C. latimanus* to seek dark environments and shelter more frequently.

As keystone species whose activities have an outsized impact on their ecosystem, increased sheltering time can affect ecosystem impacts of crayfish (Thomas et al., 2016; Fischer et al., 2022). Reduced foraging activity may limit energy acquisition by crayfish that translates into reduced growth rates and poorer condition and may reduce availability of organic materials derived from the shredding and processing of these materials by crayfish, limiting the productivity of these aquatic systems. Crayfish availability to nocturnal predators may decrease also, affecting energy transfers among trophic levels. Furthermore, the greater importance of shelter due to ALAN may increase competition for shelters, enhancing agonistic interactions and displacement of individuals where habitat complexity is low, potentially favoring larger crayfishes and more aggressive species (Usio et al., 2001; Olsson and Nystrom 2008; Chucholl et al., 2008).

However, nocturnal activity in crayfish can vary between species with some species exhibiting greater diurnal activity (Musil et al., 2010). Therefore, our results cannot be extrapolated to all crayfish species, and it is not known if *C. latimanus* daily activity cycles are dominated by nocturnal activity. However, our results support a growing body of research indicating that crayfish, invertebrates, many other taxa, and, broadly, ecosystems experience disruption and harm from the prevalence of ALAN. While providing benefits to society such as safety and security at night and supporting leisure and commercial activity at night, an analysis of trade-offs in the modern use of ALAN and proper regulation that supports aquatic biodiversity and minimizes harm should be considered (Morgan-Taylor and Kim 2016).

## Methods

*Crayfish Collection and Housing*

The variable crayfish, *Cambarus latimanus,* an abundant and prevalent crayfish in South Carolina, was captured at two small tributaries in the Lawson’s Fork Creek watershed in Spartanburg, South Carolina on 18 June 2025 and 15 July 2025. Upon capture, crayfish were immediately transported to Wofford College, where they were acclimated to the lab water source over a 30-minute period and placed into tanks for housing. Each tank was a rectangular (50.8 x 25.4 x 30.5 cm) 10-gallon tank that had 3-5 cm of cleaned gravel substrate, constant aeration, and 10-12 3.81-5.08 cm diameter PVC pipes that were cut in half lengthwise and approximately 10 cm in length to use as shelter. The mean water temperature was 20.5°C. Crayfish were fed every 3-4 days (Aqueon sinking shrimp pellets), and water changes occurred approximately once per week.

*Experiment I: Crayfish Sheltering Behavior in Varying Light Levels*

Six (15.2 x 20.3 x 20.3 cm) experimental arenas were prepared with 3-5 cm of gravel, aerated, and wrapped in a thick layer of black landscaping fabric to reduce external light and reflectivity, which can affect crayfish behavior (Rocca et al. 2024). Three treatment conditions were created – high light (~20 lux), low light (~7 lux), and no light (~1 lux) ‒ that contained two arenas each. Cardboard dividers were placed between arenas in each treatment group to isolate treatment groups and eliminate the possibility of light contamination of nearby tanks. High brightness LED 10-watt flood lights were mounted approximately 40 cm above the high light and low light treatments to provide light for each arena. To reduce the light intensity in the low light treatment, black felt was placed over the light until an intensity of 7 lx was achieved. Light intensity was measured using a Vernier LabQuest 2 Light Sensor probe. A Wyze Cam-OG 1080p HD color camera with night vision was mounted over each treatment group to record crayfish activity in two tanks simultaneously. A single crayfish was placed into each arena at 1300 hrs and acclimated in arenas for approximately 7 hours. The laboratory was darkened from 2000 hrs to 0800 hrs, and crayfish were recorded between 2000 and 0500 hrs continuously. Video footage was stored on 64 GB microSD cards as one minute duration videos, and cameras were formatted to livestream video throughout the night to monitor the experiment.

Three nights were recorded, resulting in three trials for each treatment. For each recorded night, 75 minutes of footage was selected for review of crayfish activity that consisted of 2030 – 2045 hrs, 2230 – 2245 hrs, 0030 – 0045 hrs, 0230 – 0245 hrs, 0430 – 0445 hrs. For each period, 15 1-min videos were watched and the activity displayed by the crayfish during the majority of the 1-min period was recorded. Crayfish activity was categorized as sheltered (i.e., crayfish hiding under the rock shelter), active (i.e., crayfish walking in arena), stationary (i.e., crayfish not under shelter but stationary and not near the edge of the arena), or on the edge of the tank (i.e., crayfish stationary and adjacent to the edge of the arena). Observations of crayfish along the edge of the tank were mostly stationary and flush against the edge. Thus, it was assumed that they were using the corner of the arena as shelter unless they were walking, and edge of the arena observations were considered as sheltered observations. The proportion of time spent in shelter was calculated for each crayfish. For the last trial of the experiment, crayfish that were recently captured from another stream with a reduced acclimation period (~48 hours) were used. 

*Experiment II: Crayfish Preference for Light and Dark Environments*

Six (15.2 x 20.3 x 20.3 cm) experimental arenas were prepared with 3-5 cm of gravel, constant aeration, and a layer of black landscaping fabric identical to Experiment I. Each arena was divided into two equal-sized sections created using a duct tape barrier in the middle of the tank. The barrier had a 2.5 - 4.0 cm gap between the barrier and the gravel so crayfish could move between sections. Each arena was placed into cardboard boxes and covered with a cardboard lid to create a completely dark environment. On one side of the cardboard lid, a small rectangular hole was created, and a high brightness LED 10-watt light was placed on the hole, creating a light side (~60 lx) and a dark side within the arena.

A single crayfish was placed into each arena, and crayfish acclimated in the tank for two hours and were then observed every five minutes over one hour, either being counted as in the light or dark side of the tank. Observations were made by creating a small, covered hole on the light side of the box so that observations could be made while minimizing disturbance. Crayfish that were not observed on the light side of the arena were assumed to be in the dark side of the arena. Four trials were conducted with six arenas. The first trial contained no shelter, but shelter, consisting of a 5.08 cm diameter, 10 cm long PVC pipe cut in half lengthwise, was added to each side of the arena for each successive trial. Thus, the second trial contained a PVC shelter with a light intensity of 60 lx. After observations from the first two trials, the light intensity was reduced for the third trial to 15 lx and then to 5 lx to determine if crayfish preferences for light or dark environments would change under decreasing light intensities. Thus, each trial represented four different conditions.

*Statistical Analysis*

For Experiment I, the individual crayfish (N=24) was the unit of analysis with the 75 1-minute observations from video recordings being recorded as the proportion of time spent in shelter, resulting in a single measurement of the proportion of time in shelter for each crayfish. The proportion of time in shelter was not normally distributed. Thus, a Kruskal-Wallis test ( *H(df); P-value* ) was used to compare the median proportion of sheltering among the three treatment groups. For the last trial of the experiment, crayfish that were recently captured from another stream with a reduced acclimation period (~48 hours) were used. Based upon the observation that these crayfish sheltered more often, a Mann-Whitney U test was used to compare proportion of time in shelter between the two source streams to determine if a location effect existed. Because each treatment contained two arenas during each data collection period, a Mann-Whitney U test investigated whether the proportion of time in shelter differed among the two experimental arenas to test for tank effects.

In Experiment II, all observations of a crayfish’s choice of light or dark environments over the observation period were converted to the proportion of time a crayfish spent on the dark side of the arena. A Kruskal-Wallis test compared the proportion of time on the dark side of the arena across four trials. In addition, a Kruskal-Wallis test compared medians across the six arenas used in the experiment to test for tank effects. All analyses were completed using R 4.4.2 and considered significant at *α* = 0.05.

## References

[R1] Bará Salvador, Falchi Fabio (2023). Artificial light at night: a global disruptor of the night-time environment. Philosophical Transactions of the Royal Society B: Biological Sciences.

[R2] Chucholl Christoph, Stich Hans Bernd, Maier Gerhard (2008). Aggressive interactions and competition for shelter between a recently introduced and an established invasive crayfish: Orconectes immunis vs. O. limosus. Fundamental and Applied Limnology.

[R3] Davies Thomas W., Smyth Tim (2017). Why artificial light at night should be a focus for global change research in the 21st century. Global Change Biology.

[R4] Dudgeon D, Arthington AA, Gessner MO, Kawabata Z, Knowler DJ, Leveque C, et al., Sullivan CA. 2006. Freshwater biodiversity: importance, threats, status and conservation challenges. Biological Reviews. 81(2):163–182. 10.1017/S1464793105006950 16336747

[R5] Fischer Justin R., Gangloff Michael M., Creed Robert P. (2020). The behavioral responses of 2 Appalachian crayfish to cool and warm spectrum LED lights at night. Freshwater Science.

[R6] Franke Robert, Hörstgen-Schwark Gabriele (2015). Control of activity patterns in crowded groups of male noble crayfish Astacus astacus (Crustacea, Astacidea) by light regimes: A way to increase the efficiency of crayfish production?. Aquaculture.

[R7] Gaston Kevin J., Davies Thomas W., Nedelec Sophie L., Holt Lauren A. (2017). Impacts of Artificial Light at Night on Biological Timings. Annual Review of Ecology, Evolution, and Systematics.

[R8] Gherardi F. 2002. Behaviour. Pp. 258 – 290, *In* : Biology of Freshwater Crayfish. Holdich DM (ed.). Blackwell Science. London, UK.

[R9] Jackson Kelly M., Moore Paul A. (2019). The intensity and spectrum of artificial light at night alters crayfish interactions. Marine and Freshwater Behaviour and Physiology.

[R10] Kim Wonjin, Woo Soyoung, Kim Yongwon, Kim Seongjoon (2025). Impact of rapid urbanization on flow regime and ecosystem services at seasonal scale: A case study in water conservation area along the Gyeongan River, South Korea. Science of The Total Environment.

[R11] Longcore Travis, Rich Catherine (2004). Ecological light pollution. Frontiers in Ecology and the Environment.

[R12] Loughman Zachary J., Fetzner James W. (2015). Astacology and crayfish conservation in the southeastern United States: past, present and future. Freshwater Crayfish.

[R13] Morgan-Taylor Martin, Kim Jeong Tai (2016). Regulating Artificial Light at Night: A Comparison Between the South Korean and English Approaches. International Journal of Sustainable Lighting.

[R14] Musil M, Buric M, Policar T, Kouba A, Kozak P. 2010. Comparison of diurnal and nocturnal activity between noble crayfish ( *Astacus astacus* ) and spinycheek crayfish ( *Orconectes limosus* ). Freshwater Crayfish. 17:189 – 193.

[R15] OLSSON KARIN, NYSTRÖM PER (2008). Non‐interactive effects of habitat complexity and adult crayfish on survival and growth of juvenile crayfish (
*Pacifastacus leniusculus*
). Freshwater Biology.

[R16] Reynolds J, Souty-Grosset C, Richardson A. 2013. Ecological roles of crayfish in freshwater and terrestrial habitats. Freshwater Crayfish. 19(2):197 – 218.

[R17] Rocca Stephanie M, Saldana Danielle N, Addemir Merve, Koenig Julianna A, He Bin Z, Miakotina Olga L, Eberl Daniel F (2024). Reflective environment heightens crayfish aggressive and fearful behaviors.

[R18] Sanders Dirk, Frago Enric, Kehoe Rachel, Patterson Christophe, Gaston Kevin J. (2020). A meta-analysis of biological impacts of artificial light at night. Nature Ecology & Evolution.

[R19] Sbragaglia Valerio, Breithaupt Thomas (2021). Daily activity rhythms, chronotypes, and risk-taking behavior in the signal crayfish. Current Zoology.

[R20] Tang Ziyi, Wang Pin, Li Yao, Chen Chaohui, Lou Yihan, Hu Tangao (2025). Contributions of urbanization and population growth to changes in urban flood exposure in recent decades. Sustainable Cities and Society.

[R21] Thomas John Rhidian, James Joanna, Newman Rhian Claire, Riley William D., Griffiths Siân W., Cable Jo (2016). The impact of streetlights on an aquatic invasive species: Artificial light at night alters signal crayfish behaviour. Applied Animal Behaviour Science.

[R22] Tran, N. 2025 Mar 20. Spartanburg among 10 fastest-growing metro areas in US., according to US Census Bureau. Spartanburg Herald-Journal

[R23] U.S. Census Bureau. 2025. [accessed 2025 Aug 29]. https://data.census.gov/all?q=spartanburg+county

[R24] Usio Nisikawa, Konishi Motoharu, Nakano Shigeru (2001). Species Displacement Between an Introduced and A ‘vulnerable’ Crayfish: The Role of Aggressive Interactions and Shelter Competition. Biological Invasions.

